# Ninety‐day experimental hyperthyroidism in male rats: Effects on muscle contractility, fiber composition, oxidative stress, and nerve conduction

**DOI:** 10.14814/phy2.70669

**Published:** 2025-11-27

**Authors:** João Victor Capelli Peixoto, Marcelo Cardoso Ferrari, Sarah Lethícia Lourenço, Matheus Felipe Zazula, Maria Carolina Stipp, Olair Carlos Beltrame, Alexandra Acco, Katya Naliwaiko, Fernando Augusto Lavezzo Dias, Rosalvo Tadeu Hochmueller Fogaça

**Affiliations:** ^1^ Department of Physiology Federal University of Paraná Curitiba Brazil; ^2^ Department of Cell Biology Federal University of Paraná Curitiba Brazil; ^3^ Department of Pharmacology Federal University of Paraná Curitiba Brazil; ^4^ Department of Veterinary Medicine Federal University of Paraná Curitiba Brazil

**Keywords:** diaphragm, EDL, fiber type, hyperthyroidism, oxidative stress, rodents

## Abstract

Hyperthyroidism generates a hypermetabolic state that promotes adaptations in the skeletal muscle and peripheral nerves leading to deleterious effects. However, there is a paucity of data and conflicting evidence on the influence of thyroid hormones on muscles with predominantly glycolytic fibers and on peripheral nerves. We assessed the contractility, oxidative stress, and muscle fiber composition in the extensor digitorum longus (EDL) and diaphragm, in addition to assessing the nerve conduction in isolated sciatic nerve in a Wistar rat model of chronic hyperthyroidism, induced by levothyroxine over 13 weeks. In hyperthyroid rats, the EDL increased the speed of relaxation associated with loss of muscle mass and reduction in superoxide dismutase (SOD) activity. The diaphragm gained resistance to fatigue associated with an increment in type IIa fibers at the expense of type I and IIb fibers and an increase in SOD and catalase (CAT) activities. In the sciatic nerve, latency, time to peak, and duration of compound action potential were maintained. Chronic hyperthyroidism elicits differential muscle‐specific adaptations. While the diaphragm enhances fatigue resistance and antioxidant response, the EDL undergoes atrophy and diminished antioxidant capacity. The observed changes in the sciatic nerve highlight the systemic impact of hyperthyroidism beyond direct muscle effects.

## Graphical abstract

Sequence of events leading to hyperthyroidism‐induced adaptations in skeletal muscle and sciatic nerve.

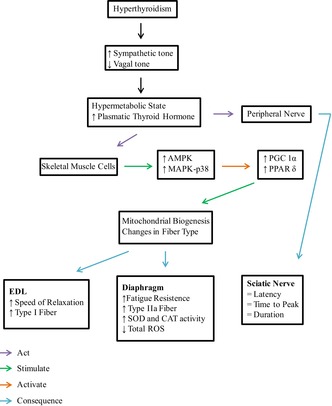

## 1 INTRODUCTION

Thyroid hormones T3 and T4 play a central role in the body by regulating biological processes such as metabolic rate, oxygen consumption, gene transcription, and protein synthesis (Mullur et al., 2014). Excess thyroid hormones or hyperthyroidism increase sympathetic tone and reduce vagal tone, creating sympathovagal imbalance (Matsumoto et al., 2022). It is associated with increased catecholamine synthesis and sensitivity to catecholamines in tissues (Burggraaf et al., 2001), generating a hypermetabolic state.

Common symptoms of hyperthyroidism in young patients are associated with increased sympathetic activity, such as tremor, tachycardia, and palpitations, but are also related to changes in muscle fiber types (Bloise et al., 2018), leading to weakness, myalgia, fatigue, and cramps (Duyff et al., 2000; Kung, 2007). The extent and severity of symptoms are related to the degree and duration of the disease (Kung, 2007), being observed in elderly patients the development of thyrotoxic myopathy, a neuromuscular disorder that results in muscle wasting, reduced strength, weakness, and fatigue (Everts, 1996; Kung, 2007).

There is limited data on the effects of hyperthyroidism on nerve conduction. There are reports linking thyrotoxic peripheral neuropathy to progressive weakness, that may eventually progress to flaccid paraplegia (Al‐Wahaibi et al., 2017; Pal et al., 2014). In a cross‐sectional study in patients with hyperthyroidism, 10.7% presented abnormal results in nerve conduction studies, that were mostly due to axonal neuropathy (Singh et al., 2024). Another case–control study also demonstrated axonal neuropathy in this population (Faraz et al., 2021).

Several studies have demonstrated that increases in plasma thyroid hormone concentrations significantly alter morphological and contractile parameters in slow‐twitch muscles, and smaller effects are observed in fast‐twitch muscles (Fitts et al., 1984; Nicol & Bruce, 1981; Singh et al., 2004; Yamada et al., 2007).

T3 rapidly and non‐genomically stimulates the activity of AMP‐activated protein kinase (AMPK) and p‐38 mitogen‐activated protein kinase (MAPK‐p38) signaling pathways in muscle cells (Irrcher et al., 2008). AMPK and MAPK‐p38 phosphorylate and activate peroxisome proliferator‐activated receptor γ coactivator‐1α (PGC‐1α) (Ji, 2015; Kramer & Goodyear, 2007). PGC‐1α stimulates transcription factors such as peroxisome proliferator‐activated receptor (PPAR), nuclear respiratory factor (NRF)‐1 and NRF‐2, mitochondrial transcription factor (Tfam), and myocyte‐enhancing factor (MEF) 2 (Ji, 2015; Nakamura et al., 2014). PPARδ is the most abundant isoform in skeletal muscle, and its activation is the primary mechanism for increasing free fatty acid utilization during stressful times (Nakamura et al., 2014), such as during chronic hyperthyroidism.

Hyperstimulation of the aforementioned signaling pathways modulates the metabolic characteristics of the muscle, resulting in mitochondrial biogenesis and changes in fiber type (Irrcher et al., 2008; Johnson et al., 1983; Nicol & Bruce, 1981). These metabolic modulations allow the skeletal muscles to adapt to hypermetabolism induced by hyperthyroidism. Notwithstanding, muscle biopsy reveals atrophy in type I and II muscle fibers, fatty infiltration, muscle fiber necrosis, and lymphocytic infiltration (Ramsey, 1966). In respiratory muscles, a reduction in maximum strength and dyspnea have been reported (Goswami et al., 2002; Yamada et al., 2007).

Thyroxine (T4) treated rats are an animal model used for the study of hyperthyroidism; however, evidence regarding contractile parameters of fast‐twitch muscles and changes in fiber type is conflicting, and the impact on nerve conduction and on oxidative stress is scarce.

In this study, we aimed to evaluate in 90 days T4‐treated rats the skeletal muscle contractility, the fiber type, and the tissue oxidative stress using the extensor digitorum longus (EDL) and diaphragm muscles, as well as the nerve conduction assessed in isolated sciatic nerve.

## 2 METHODS

### 2.1 Animals

All experimental protocols used in this study were approved by the Animal Experimentation Ethics Committee of the Biological Sciences Sector at the Federal University of Paraná (license number: CEUA/UFPR 1547) and were conducted in compliance with the National Institutes of Health's Guide for the Care and Use of Laboratory Animals (CONCEA/MCTI). Thirty‐two male Wistar rats, weighing 250–300 g were provided by the Experimental Animal Center of the UFPR. Animals were kept in cages under controlled conditions of temperature and a light–dark cycle of 12 h, with free access to food (Nuvilab CR‐1, Quimtia, Brazil) and water. Environmental enrichment was performed using polyvinyl chloride (PVC) pipes and paper napkins.

### 2.2 Experimental model of hyperthyroidism

Rats were randomly divided into two groups: Control (*n* = 16) and hyperthyroidism (HY, *n* = 16). HY animals received a daily dose of levothyroxine sodium (Puran T4, Sanofi, Brazil. Catalogue code: 79712) diluted in water, administered by gavage. T4 supplementation was performed five times a week for 13 weeks (Fidale et al., 2018). To ensure that the animals' adaptation to the drug effects would be gradual, the dosage was increased every 2 weeks. In the first 2 weeks, 12 μg/100 g was administered, followed by 16 μg, 18 μg, 20 μg and in the final 5 weeks the dose was 25 μg/100 g of body weight. Control animals were treated with water for the same period and route of administration. The animals were weighed weekly for dosage adjustment. At the end of the experimentally induced hyperthyroidism period, the animals were anesthetized with an intraperitoneal injection of ketamine (80 mg kg^−1^) and xylazine (20 mg kg^−1^) and euthanized by exsanguination. Blood samples were collected in heparinized tubes and centrifuged at 500*g* for 5 min. The plasma was stored at −80°C for later analysis.

### 2.3 Plasma analysis

To confirm the HY model in rats, the plasma samples were subjected to total T3, total T4, free T4, and cardiac troponin I measurements using automated equipment (Vitros XT 7600 Integrated System from Ortho Clinical Diagnostics). All measurements were made using chemiluminescence immunoassay (Ortho Clinical Diagnostics plasma hormone test kit, Brazil) (Immunoassay kit catalogue codes: tT3: 132528, tT4: 8744468, fT4: 1387000, and trop. I: 6844436).

### 2.4 Muscle and sciatic nerve dissection

Immediately after euthanasia, the chest cavity was opened, and the heart and entire diaphragm were removed. Next, the two EDLs were removed from the posterior members, followed by the removal of a 5 cm portion of the sciatic nerve from the sciatic notch to the anterior tibialis muscle on the right side. Immediately after removal, the heart was washed and weighed on a precision scale, and the muscles and nerves were placed in a Petri dish containing Ringer's solution of the following composition: NaCl 110 mM, KCl 4 mM, CaCl_2_ 2 mM, MgCl_2_ 2 mM, TRIZMA 10 mM and glucose 11 mM, and adjusted to pH 7.4 with NaOH or HCl, gassed with pure oxygen and maintained at 37°C. The left half of the diaphragm and right EDL were used for contractility experiments (Singh et al., 2004). The right half of the diaphragm and left EDL were stored at −80°C and later used for oxidative stress and histology experiments.

### 2.5 Assessment of contractility in isolated muscle

On the left portion of the diaphragm, a 10 mm thick strip was dissected from the costal portion towards the tendon portion. As described by Peixoto et al. (2020) the costal end of the diaphragm was fixed to a stationary clip, while the tendinous portion was attached to a force transducer (Fort 10 WPI, Transduction Laboratories Co.) to measure contractions under isometric conditions. Electrical stimulation was performed directly through two platinum electrodes positioned along the muscle in the bath chamber, which were connected to an electrical stimulator. As described by Ryall et al. (2002) the right EDL was prepared in a similar manner.

Each muscle was stretched through a micromanipulator coupled to a stationary clip until the optimum muscle length (Lo) was determined from a single electrical stimulus of different lengths. The length at which a single contraction was maximum was considered Lo.

Immediately after determining Lo, muscles were electrically stimulated with a suprathreshold voltage of 24 V with a one millisecond pulse and a standard frequency of 0.5 Hertz for 7 min to evaluate contractile endurance. Parameters such as maximum isometric twitch force (Tmax), maximum speed of force development (+dF/dt), and maximum speed of force decrease (–dF/dt) were evaluated. At the end of the seventh minute, to evaluate endurance resistance, the stimulus frequency was increased to 100 Hz, where the maximum tetanic force (Fmax) and resistance to fatigue were recorded. Resistance to fatigue was calculated as the time required to reduce Fmax to 50% of its value. At the end of each experiment, muscle length at Lo was measured using a caliper and the muscle was weighed on a precision scale.

### 2.6 Cross‐section area (CSA) calculation and data normalizing

For the diaphragm, CSA was calculated using the formula: muscle weight/(muscle length × muscle density), assuming a density of 1.056 g/cm^3^ (Vrabas et al., 1999). For the EDL, the formula used was as follows: muscle weight/[muscle density × (muscle length × constant)], assuming a density of 1.06 g/cm^3^ and a constant of 0.44 (Ryall et al., 2002). Tmax, +dF/dt, –dF/dt, and Fmax were normalized by CSA. The results are presented as N/cm^2^. The data were collected using a PowerLab 4/30 (AD Instrument) acquisition system and subsequently analyzed using Lab Chart version 7.3.7 software.

### 2.7 Determination of muscle fiber type

The diaphragm and EDL stored at −80°C were transferred to the cryostat chamber at −25°C (Leica Wetzlar, Germany) and fixed to platinum with Tissue‐Tek® compound. Subsequently, 7 μm cross‐sections of the muscles were obtained, fixed on glass slides, and intended for histoenzymological techniques. For the fiber‐type profile identification of the diaphragm and EDL, one slide with six sections was obtained per animal. This slide was subjected to the nicotinamide adenine nicotinamide‐tetrazolium reductase (NADH‐TR) reaction (Dubowitz & Brooke, 1973). The obtained material was used to analyze the oxidative and glycolytic metabolism of the three types of fibers.

For histoenzymological analysis, the slides were photographed under a light microscope (Carl Zeiss™ Primo Star™), attached to a camera (Carl Zeiss™ AxioCam ERc 5 s) in the program ZEN 3.1 (Carl Zeiss™). Ten microscopic fields were used for the analysis performed at 200x magnification (lens Zeiss‐Primo, Plan‐ACHROMAT). This material was analyzed using Image‐Pro Plus software (version 6.0; Media Cybernetics, Rockville, MD, USA).

### 2.8 Oxidative stress

The diaphragm and EDL tissue samples (0.1 g) were homogenized in potassium phosphate buffer (pH 6.5, 1:10 w/v) and centrifuged at 10000*g* for 20 min at 4°C. The resulting homogenate was used to assess the reduced glutathione (GSH) levels (Sedlak & Lindsay, 1968), whereas the supernatant was used to determine superoxide dismutase (SOD) activity (Gao et al., 1998), catalase (CAT) activity (Aebi, 1984), total ROS levels (Keston & Brandt, 1965), and lipid peroxidation (LPO) levels (Jiang et al., 1991). The results are expressed according to the amount of tissue or total protein in the samples, as determined using the Bradford (1976) method (Bradford reagent, Sigma‐Aldrich catalogue code: B6916).

### 2.9 Sciatic nerve conduction parameters

After removal, the nerve was immediately transferred to a nerve chamber (MLT016/B, AD Instrument) filled with Ringer's solution at 37°C, kept immersed and suspended on stainless steel electrodes. The sciatic nerve was stimulated with square pulses at its proximal end. The cathode and the anode were separated by 0.5 cm, with the cathode positioned closest to the recording electrodes, which were placed 3 cm away (Medler, 2022). The stimulus parameters were as follows: pulse duration of 1 ms, stimulus intensity of 6 V, and a frequency of 0.5 Hz (Yildirim et al., 2022). Recordings were made using a Power Lab 26T acquisition system (AD Instrument) and data was analyzed using Lab Chart software version 7.3.7. The average of 10 compound action potentials was calculated for each isolated nerve. Latency, time to peak, amplitude, duration, response area, and slope were evaluated for the upward deflection of the compound action potential (normally biphasic).

### 2.10 Statistical analysis

Results are expressed as mean values ± standard deviation (SD). To analyze Tmax, +dF/dt, and –dF/dt, where the variables were T4 supplementation (HY vs. Control) and contractions over time, a two‐way ANOVA followed by Bonferroni's post hoc test was used. Student's *t*‐test was used for analysis, comparing two groups. The Shapiro–Wilk test was used to test data normality. When the normality test failed, the Mann–Whitney rank sum test was used. GraphPad Prism 8 System (San Diego, CA, USA) was used for statistical analysis and plotting. Statistical significance was set at *p* < 0.05.

## 3 RESULTS

After 13 weeks of experimentally induced hyperthyroidism, it was observed that HY (*n* = 16) body weight showed no difference when compared to Control (*n* = 16). However, the heart weight increased 12.6%, and the heart weight/body weight ratio increased 14.8% in the HY group. The diaphragm muscle weight (*n* = 6) did not differ among groups, but the EDL weight (*n* = 6) decreased by 19.6% in the HY group. The plasma thyroid hormones and cardiac troponin I dosage showed a boost of 230.9% for free T4 (*p* = 0.002), 104.9% for total T3 (*p* = 0.009), 238.6% for total T4 (*p* < 0.001), and 125.4% for cardiac troponin I (*p* = 0.009), confirming the HY model in the rats that received levothyroxine. The results are presented in Table 1.

**TABLE 1:** Animal, heart, and muscle weights and plasma dosages.

	Control	Hyper	*p* Value
Body weight (g)	454.25 ± 42.12	443.83 ± 25.89	0.473
Heart weight (g)	1.59 ± 0.17	1.79 ± 0.21	0.021
B.w./H.w. ratio (g)	3.52 ± 0.45	4.04 ± 0.53	0.019
Diaphragm weight (g)	0.22 ± 0.043	0.22 ± 0.041	0.794
EDL weight (g)	0.24 ± 0.024	0.20 ± 0.015	0.002
Free T4 (ng/dL)	1.28 ± 0.20	4.23 ± 1.85	0.002
Total T3 (ng/mL)	0.71 ± 0.048	1.46 ± 0.69	0.009
Total T4 (μg/dL)	3.46 ± 0.39	11.72 ± 5.09	<0.001
Cardiac troponin I (ng/L)	1.90 ± 0.51	4.28 ± 2.00	0.009

*Note*: Values are expressed as mean ± SD.

### 3.1 Contractile measurements

In the diaphragm, Tmax, +dF/dt, –dF/dt, and Fmax did not differ between the groups (data not shown). However, a significant increase of 52.3% in time to fatigue was observed in HY (*n* = 6) [60.0 ± 15.05 vs. 39.4 ± 7.89 sec (*p* = 0.022)] compared to Control (*n* = 6).

**FIGURE 1:**
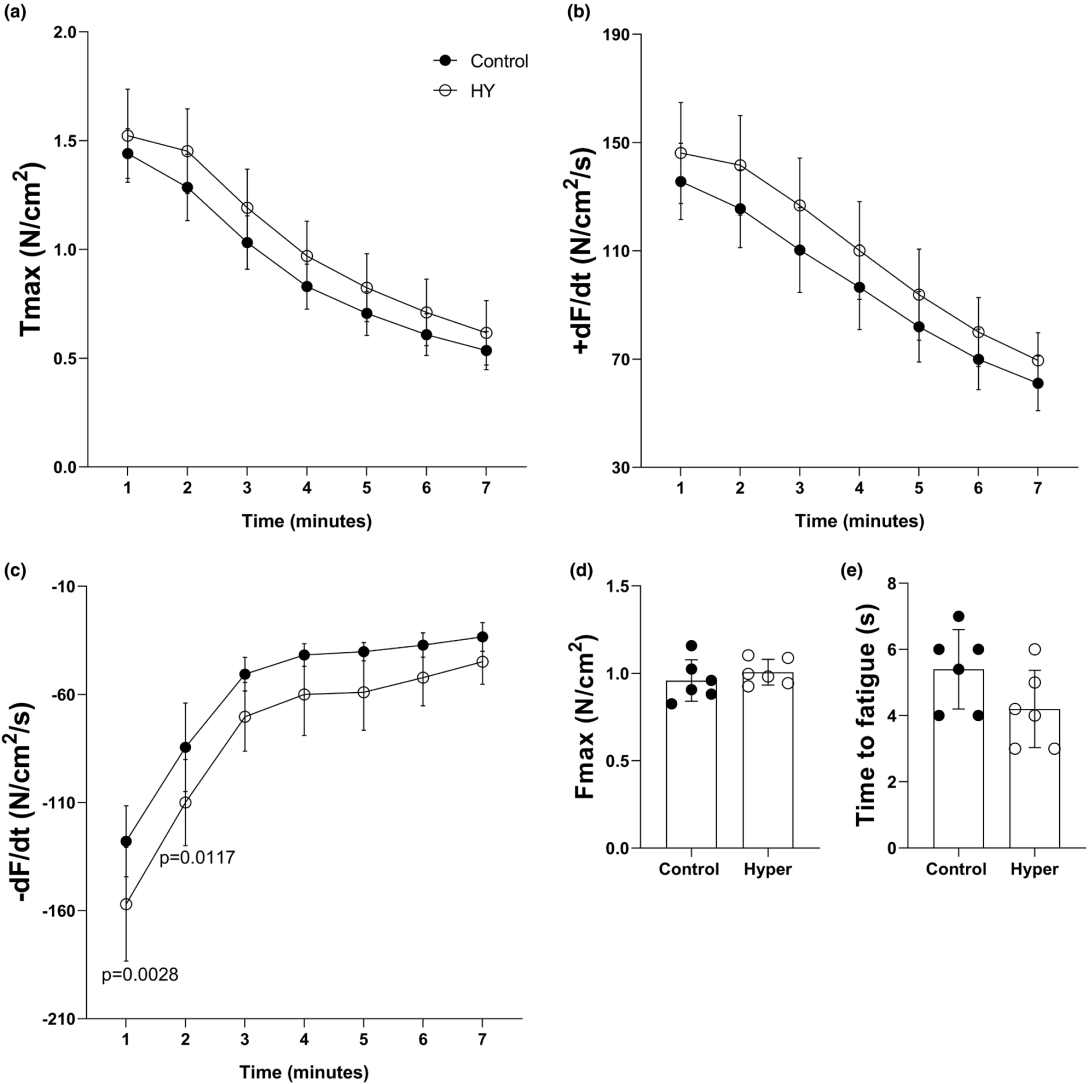
Contractile parameters from the EDL muscle: (a) T_max_, (b) +dF/dt, (c) –dF/dt, (d) F_max_, and (e) time to fatigue. Control, *n* = 6; HY, *n* = 6.

In the EDL, Tmax, +dF/dt, Fmax, and time to fatigue did not differ between the groups. –dF/dt demonstrated a boost of 23.6% in the first [−157.00 ± 26.28 vs. –127.94 ± 16.41 N/cm^2^/s (*p* = 0.0028)] and 30.9% in the second minute [−110.03 ± 19.94 vs. –84.43 ± 20.47 N/cm^2^/s (*p* = 0.0117)] in the HY group (*n* = 6) compared to the Control group (*n* = 6). The complete data are shown in Figure 1.

### 3.2 Proportion of muscle fiber type

**FIGURE 2:**
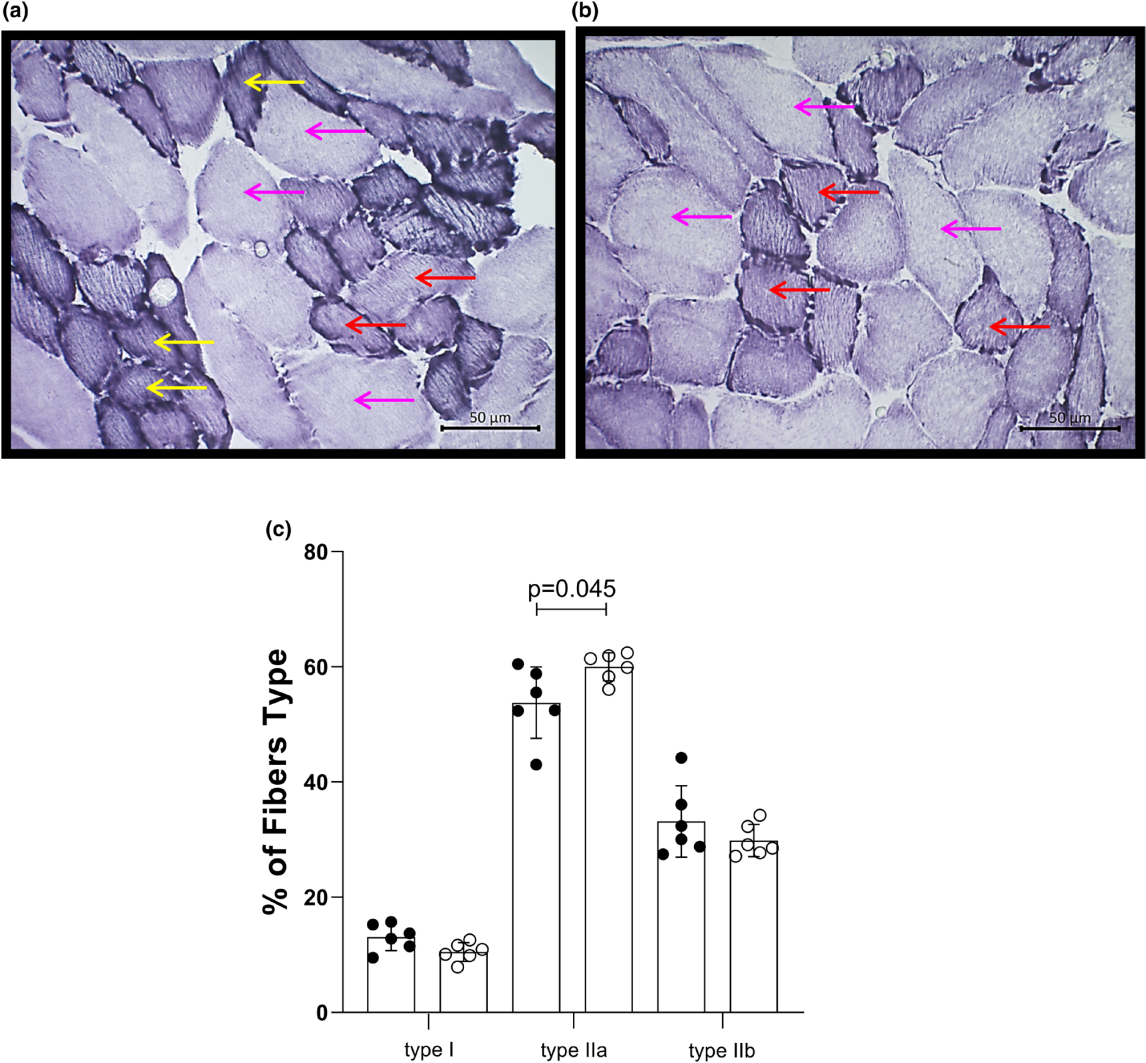
Transverse cryosections at 7 μm from rat diaphragm submitted to NADH‐TR reaction for fiber typing. (a) Control, (b) HY, (c) percentage of fiber types. The yellow arrow indicates type I fiber, the red arrow indicates type IIa fiber, and the pink arrow indicates type IIb fiber. Control, *n* = 6; HY, *n* = 6.

Represented in Figure 2a–c, HY (*n* = 6) diaphragm muscle demonstrated a shift to type IIa fibers at the expense of type I and IIb fibers with preserved muscle weight (Table 1). The percentage of fibers compared to Control (*n* = 6) was as follows: type IIa fibers 60.00 ± 2.45% versus 53.78 ± 6.21% (*p* = 0.045), type I 10.51 ± 1.63% versus 13.07 ± 2.34% (*p* = 0.052), and IIb fibers 29.49 ± 2.80% versus 33.15 ± 6.20% (*p* = 0.259).

**FIGURE 3:**
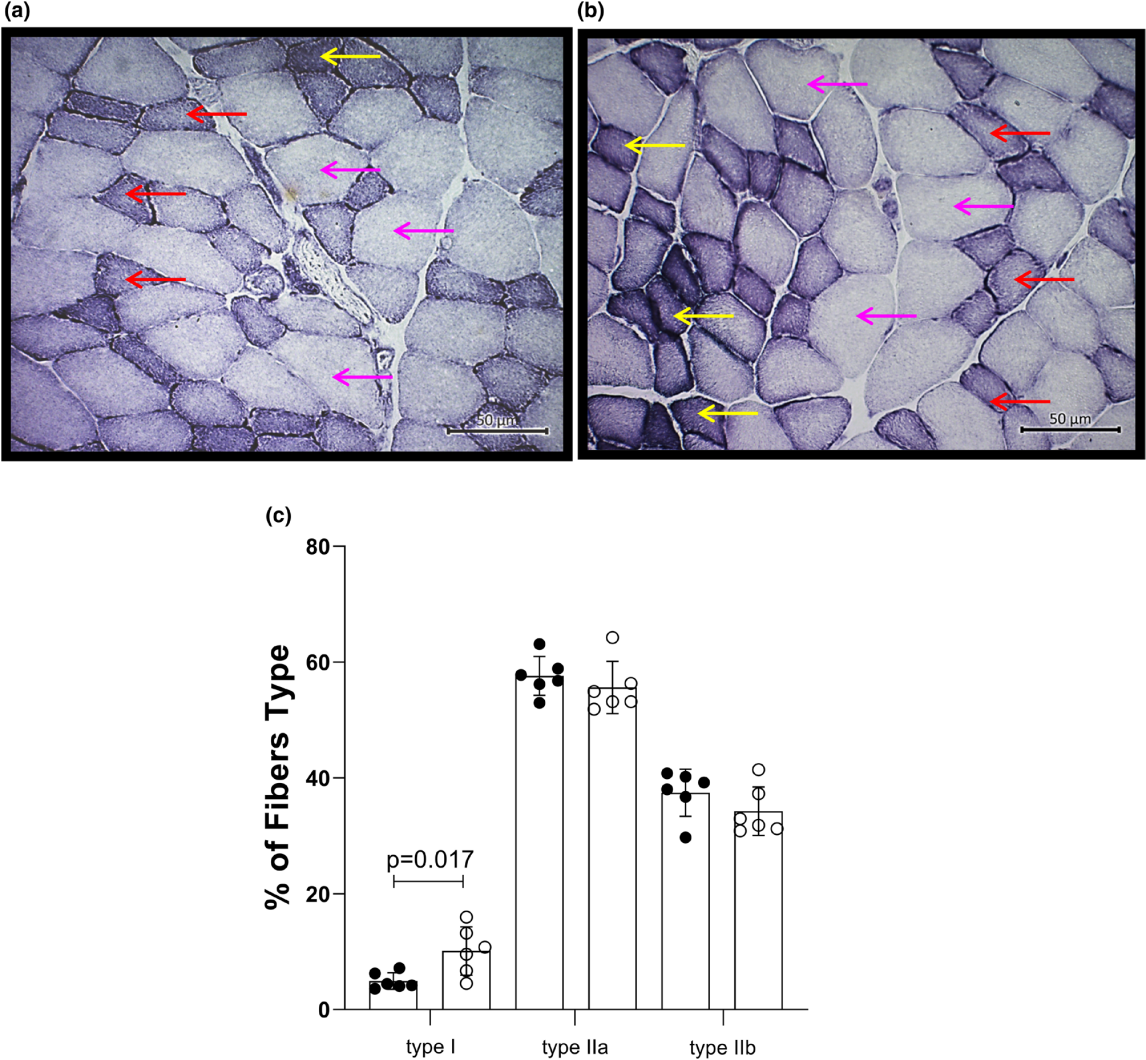
Transverse cryosections at 7 μm from rat EDL submitted to NADH‐TR reaction for fiber typing. (a) Control, (b) HY, and (c) percentage of fiber types. The yellow arrow indicates type I fiber, the red arrow indicates type IIa fiber, and the pink arrow indicates type IIb fiber. Control, *n* = 6; HY, *n* = 6.

In Figure 3a–c, EDL muscle from HY (*n* = 6) demonstrated an increment in type I fibers associated with a significant decrease in muscle weight (Table 1). The percentage of fiber compared to Control (*n* = 6) was as follows: type I fiber 10.11 ± 4.18% versus 4.94 ± 1.42% (*p* = 0.017), type IIa 55.63 ± 4.50% versus 57.62 ± 3.36% (*p* = 0.405), and type IIb 34.26 ± 4.21% versus 37.44 ± 4.06% (*p* = 0.212).

### 3.3 Oxidative stress parameters

In the diaphragm, HY (*n* = 6) showed no significant difference in GSH (*p* = 0.786) and LPO levels (*p* = 0.815). SOD and CAT activities demonstrated an increment of 32.9% (*p* = 0.024) and 58.4% (*p* = 0.028), respectively. Total ROS decreased by 24% (*p* = 0.019) compared to the control (*n* = 6).

**FIGURE 4:**
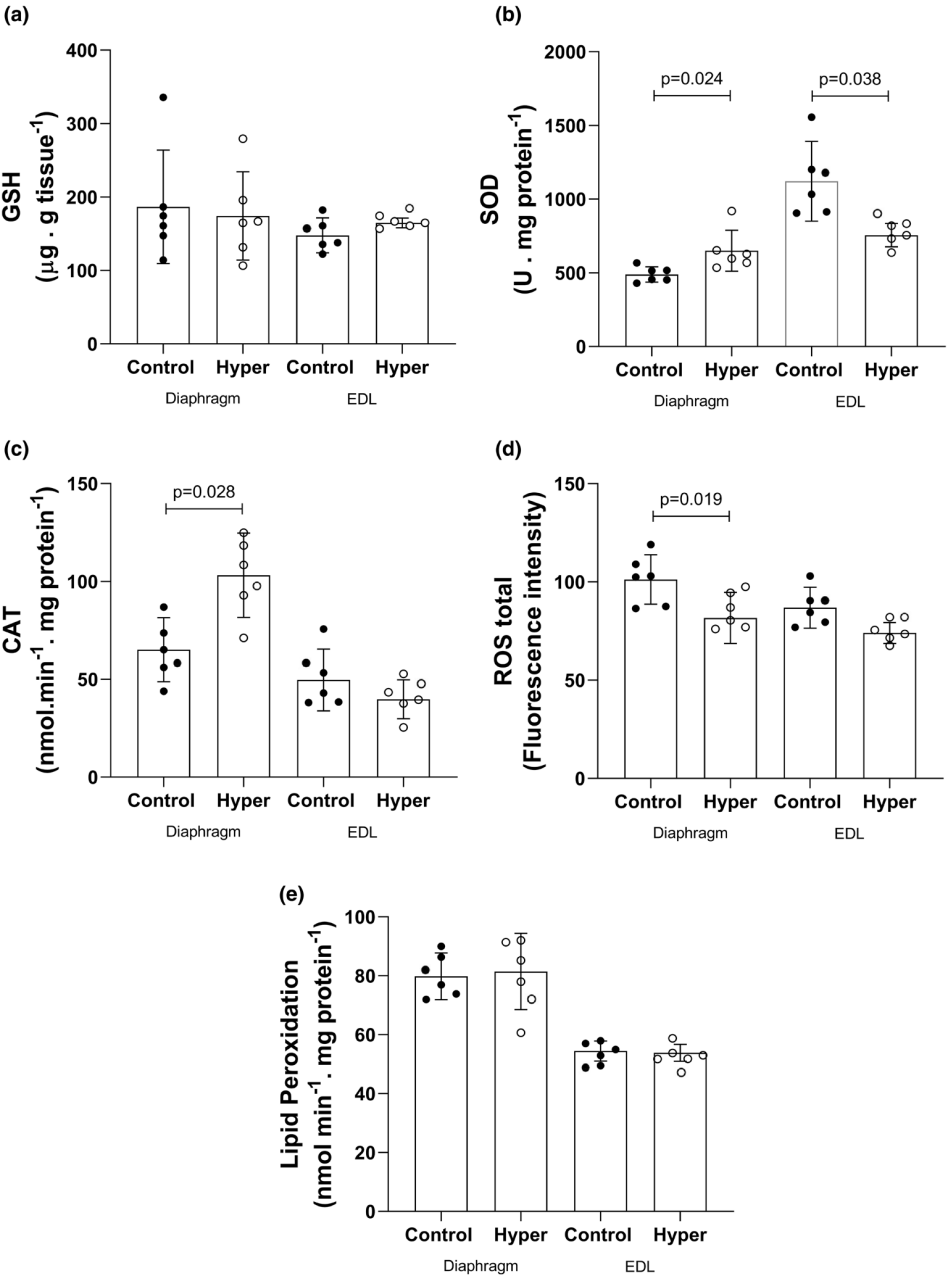
Oxidative stress parameters. (a) Reduced glutathione levels (GSH), (b) superoxide dismutase activity (SOD), (c) catalase activity (CAT), (d) total tissue reactive oxygen species (ROS), and (e) tissue lipid peroxidation (LPO). Diaphragm Control and HY, *n* = 6; EDL Control and HY, *n* = 6.

In the EDL, HY (*n* = 6) showed no difference in GSH level (*p* = 0.319), CAT activity (*p* = 0.269), total ROS levels (*p* = 0.096), and LPO (*p* = 0.877). SOD activity decreased by 48.3% (*p* = 0.038) when compared to the Control (*n* = 6). The complete data are shown in Figure 4.

### 3.4 Isolated sciatic nerve

While latency, time to peak, and duration were unchanged between groups, the HY group (*n* = 8), exhibited significant reductions in amplitude, response area, and slope relative to Control (*n* = 8). The complete data are presented in Table 2.

**TABLE 2:** Isolated sciatic nerve conduction parameters.

	Control	Hyper	*p* Value
Latency (ms)	0.75 ± 0.0	0.75 ± 0.0	1.0
Time to peak (ms)	1.87 ± 0.13	1.80 ± 0.10	0.119
Duration (ms)	3.64 ± 0.13	3.53 ± 0.13	0.067
Response area (V.s)	0.00284 ± 0.00038	0.00236 ± 0.00056	0.023
Amplitude (V)	1.51 ± 0.20	1.28 ± 0.29	0.036
Slope (V/s)	1178.73 ± 158.65	1001.67 ± 233.22	0.041

*Note*: Values are expressed as mean ± SD. Control, *n* = 8; HY, *n* = 8.

## 4 DISCUSSION

To the best of our knowledge, this is the first study to induce hyperthyroidism in rats for 90 days and evaluate its effects on nerve conduction in isolated sciatic nerve and contractility, focusing on changes in fiber type and in oxidative stress parameters, in the diaphragm and EDL.

The experiments evaluating diaphragm contractility in this study showed no differences in contractile parameters measured; despite that, a 52.3% gain in fatigue resistance was observed in the HY group. Only one report assessed fatigue resistance before and found no differences; however, using hamsters as a model and a different protocol to determine fatigue development (Singh et al., 2004) what precludes direct comparison to our study. Regarding contractile parameters in diaphragm muscle, the available reports present divergent results. Miyashita et al. (1992) treated dogs with 0.6 g/kg/day of T4 for 4 weeks and described a 63% increase in –dF/dt in the crural portion but no differences in the costal portion. In contrast, Singh et al. demonstrated in hamsters treated with 0.24 mg/T4/day for 2 months, a gain in Tmax and in +dF/dt and no changes in –dF/dt. Yamada et al. (2007) treated rats with 300 μg/day of T3 for 21 days and demonstrated a 54% decrement in Tmax and 25% in Fmax. +dF/dt and –dF/dt remained unchanged. The differences observed among studies may be due to different hormonal levels reached in plasma as a consequence of the thyroid hormone supplementation as well as the duration of induced hyperthyroidism. Additionally, it may be due to species‐specific skeletal muscle adaptations.

Diaphragm ATPase histology showed that HY animals' muscle decreased by 19.6% in type I fibers and 11.1% in type IIb fibers. However, type IIa fibers increased by 11.6% compared to Control. Similar to our findings, Johnson et al. (1983) treated rats with 10 μg/kg/day of T3 for 12 weeks and demonstrated that HY animal muscle composition decreased by 11.3% in type I fibers and 26.5% in type IIb fibers but increased by 22.7% in type IIa fibers. Nevertheless, Canepari et al. (1998) treated female rats with 0.2 mg/kg/day for 15 days and observed that the muscle composition was reduced by 68.2% in type I fibers and increased by 13.7% in IIa fibers and 29.4% in IIb fibers. The observed changes in fiber type were congruent among studies, indicating changes compatible with an increment in IIa fibers and a decrement in type I fibers.

Diaphragm tissue oxidative stress measurements demonstrated no change in lipid peroxidation (LPO) rates associated with a 32.9% increase in SOD and a 58.4% increase in CAT activity. The 24% decrease in total ROS indicates that the antioxidant system meets the tissue metabolic demand. In 2007, Yamada et al. (2007) reported a 104% increase in carbonyl content in the myofibrillar proteins of the diaphragm, which is indicative of oxidative stress.

Thus, in the diaphragm muscle, the results of tissue oxidative stress and myosin ATPase histology justify the results of muscle contractility. No changes were observed in contractile parameters due to oxidative control, and no significant changes were observed in the MHC I or IIb isoforms. This increase in fatigue resistance may be attributed to an increase in MHC IIa protein content associated with a favorable environment to manage oxidative stress.

EDL contractility measurements demonstrated no changes in Tmax, +dF/dt, Fmax, and fatigue resistance. However, the relaxation speed (–dF/dt) was augmented by 23.6% in the first minute and 30.9% in the second minute of experimentation in the HY group. The available data presented opposite results. Nicol & Bruce (1981) treated rats for one to 6 weeks with 200 μg/kg/day of T3 and observed a weekly progressive increase in –dF/dt, but without significance. Fatigue resistance was increased. Fitts et al. (1984) fed female rats with chow containing 3 mg/T4 and 1 mg/T3 for 6 weeks and described no change in the contractile parameters measured. Singh et al. (2004) reported an increase in +dF/dt, and no changes in –dF/dt and in Tmax.

EDL ATPase histology demonstrated that the HY muscle had a significant increase in type I fibers (104.6%), but a decline of 3.6% in type IIa fibers and 9.3% in IIb fibers when compared to control. Nicol and Bruce (1981) observed an increment of 19% in IIa fibers and a diminution of 69.2% in type I fibers and 28.2% in IIb fibers after 6 weeks of T3 treatment. Nevertheless, Larsson et al. (1994) treated young (3–6 months old) and old (20–24 months old) rats with 300 μg/kg/every other day of T3 for 4 weeks, and revealed a decrement of 3.2% in type IIb fibers and an augmentation of 30.3% in type I fibers and 4.3% in type IIa fibers in old animals. These changes were not present in young animals. All studies reported some degree of muscle atrophy. We did not perform serial assessments of muscle fiber composition at the time points previously investigated in the literature (i.e., 4–8 weeks), but the fact that the T4 treatment lasted 13 weeks could have caused a higher degree of atrophy, predominantly of type II fibers, which caused an increment in the proportion, not in number, of type I fibers. Additionally, animals grown older under T4 supplementation and age may become an additional component influencing changes in fiber type, as observed by Larsson et al. (1994).

EDL tissue oxidative stress data demonstrated no changes in GSH, CAT, total ROS, or LPO rates. SOD activity was reduced by 48.3%, indicating the absence of oxidative stress in the EDL muscle. In agreement with our results, Asayama et al. (1987) treated rats with T4 diluted in drinking water (0.0012%) for 4 weeks and observed that there was no change in SOD activity or LPO rates. Glutathione peroxidase (GPX) activity decreased and was accompanied by a reduction in catalase (CAT). The author discusses that in hyperthyroidism, free radicals are produced in complexes I and III in the mitochondrial electron transport chain, and as EDL is predominantly composed of glycolytic fibers, oxidative damage was not observed.

Contractile parameters, such as +dF/dt and –dF/dt are determined by the predominant MHC in the muscle (Fitts & Widrick, 1996) and SERCA isoforms (Periasamy et al., 2017). Although in our study the population of type I fibers increased by 104.6% in the EDL of HY animals, this number represents 10.1% of the total fibers versus 4.9% in the Control group. Arai et al. (1991) treated rabbits with T4 (200μg/kg/day) during 4 or 8 days and measured the expression of SERCA2 (slow‐twitch fiber) and SERCA1 (fast‐twitch fiber) in the plantar muscle (89% type II fiber) (Maxwell et al., 1992) and demonstrated that after 4 days an increment of 164% in SERCA2 and 201% in SERCA1. After 8 days, a gain of 169% was observed in SERCA2 and 239% in SERCA1.

Thus, the slight increase in –dF/dt observed in our study may be attributed to data normalization, which considers the muscle weight, since non‐normalized values showed no significant differences. The EDL muscles from the HY group presented a 19.6% weight reduction, as was also observed by Nicol & Bruce (−11.4%) and Fitts et al. (−17.6%). However, SERCA expression may have a contribution to this outcome.

Previous research concerning the activity of signaling pathways in hyperthyroid animals highlights pathways involved in energy metabolism regulation, transition of fiber types, and protein degradation pathways. Irrcher et al. (2003) treated rats with 0.4 mg/kg/day of T3 for 5 days and observed a gain of 50% in PGC‐1α expression in the plantar muscle (94% type II fiber) (Delp & Duan, 1996). The author discusses that PGC‐1α is a regulator of fiber type transition in skeletal muscle. Park et al. (2002) fed rats with chow containing 3 mg/T4 and 1 mg/T3 for 21 days and demonstrated in white quadriceps (94% type II fiber) (Delp & Duan, 1996) a reduction in the content of malonyl‐CoA associated with an increase in the content of acetyl‐CoA carboxylase (ACC) and AMPK. It is known that AMPK phosphorylates and inactivates ACC, resulting in a decrease in malonyl‐CoA, which reduces the inhibition of carnitine palmitoyltransferase, allowing fatty acid oxidation to occur (Miklosz et al., 2012; Park et al., 2002). Yet, O'Neal et al. (2009) treated rats with 100 μg/100 g of T3 for 7 days and demonstrated that ubiquitin, MuRF‐1 and MAFbx mRNA levels were increased and associated with a 30% increase in the rate of protein degradation and a 17% reduction in EDL weight. In this way, it is expected that an increment in muscle fibers containing mitochondria to meet the rise in β‐oxidation stimulus and decrements in muscle weight due to atrophy is supposed to be observed.

The involvement of peripheral nerves in hyperthyroidism receives little attention and reports are scarce. Specifically in the sciatic nerve of hyperthyroid rats there is no functional data. Similar to our findings, Miyashita et al. (1992) reported in dogs no changes in the latency of phrenic and femoral nerves. Also, Duyff et al. (2000) conducted electrodiagnostic studies in hyperthyroid patients and reported no changes in peroneal and medial motor nerve parameters; notwithstanding, they observed signs of sensorimotor axonal neuropathy in about 20% of the patients with hyperthyroidism. Akgul et al. (2024) performed electrodiagnostic studies in hyperthyroid patients and reported a reduction in amplitude of 30.5% in the median nerve and 27.8% in the ulnar nerve, compared to controls; as well as the same percentage of sensorimotor axonal neuropathy (about 20% of the patients). Additionally, Singh et al. also reported in hyperthyroid patients that presented abnormal results in nerve conductions studies, most of the changes were due to axonal neuropathy.

Collectively, these findings suggest that axonal dysfunction likely contributes to the clinical symptoms observed in this population. Our in vitro data, using isolated nerves, specifically show evidence of axonal dysfunction in the HY group, with significant reductions in amplitude (18%), response area (20.3%), and slope (17.7%) compared to the Control group. A limitation of the current study is the lack of peripheral nerve histomorphometry to determine if the observed functional changes correlate with morphological alterations, such as a shift in axon size or axonal loss. Future research should investigate these specific morphological changes induced by chronic hyperthyroidism and explore other potential mechanisms for nerve dysfunction, such as ion channel expression.

Other limitations of the study design were that it used only male rats, which do not allow demonstrating a potential difference between sexes in the pathophysiological aspects of thyrotoxic myopathy.

## 5 CONCLUSION

The chronic hyperthyroidism in rats elicits differential muscle‐specific adaptations. While the diaphragm demonstrated enhanced fatigue resistance and a robust antioxidant response, the EDL undergoes atrophy and exhibits altered relaxation kinetics with a diminished antioxidant capacity. The observed changes in the sciatic nerve further highlight the systemic impact of hyperthyroidism beyond direct muscle effects. These findings contribute to a comprehensive understanding of the complex pathophysiology of hyperthyroidism on diverse physiological systems.

## AUTHOR CONTRIBUTIONS

JVCP: Participated in contractile, oxidative stress and histology experiments, coordinated the data analysis, and contributed to the writing of the manuscript. MCF: Participated in nerve conduction experiments and data analysis. SLL: Participated in oxidative stress and histology experiments, and organized the experimentation days. MFZ: Participated in histology experiments and data analysis. MCS: Participated in oxidative stress experiments and data analysis. OCB: Coordinated and participated in blood analysis experiments. AA: Coordinated the oxidative stress experiments and contributed to the writing of the manuscript. KN: Coordinated the histology experiments and reviewed the manuscript. FALD: Designed the research plan, data analysis and reviewed the manuscript. RTHF: Designed the research plan, organized the study and contributed to the writing of the manuscript.

## FUNDING INFORMATION

This work was funded by CAPES/Brasil, Award No: 88887.923446/2023‐00, Programa 13179—PDPG—Pós‐Doutorado Estratégico.

## CONFLICT OF INTEREST STATEMENT

The authors declare that they have no conflict of interest, financial or otherwise.

## ETHICS STATEMENT

The research was carried out in accordance with the National Institutes of Health's Guide for the Care and Use of Laboratory Animals (CONCEA/MCTI). The Animal Experimentation Ethics Committee of the Biological Sciences Sector at the Federal University of Paraná approved all procedures in this study.

## Data Availability

Data will be provided upon reasonable request to the corresponding author.
